# Supplementary material for: Effectiveness of Respiratory Muscle Training for Pulmonary Function and Walking Ability in Patients with Stroke: A Systematic Review with Meta-Analysis
Source: Int J Environ Res Public Health. 2020 Jul 24;17(15):5356. doi: 10.3390/ijerph17155356 (PMC7432552; doi:10.3390/ijerph17155356)
Supplement: Supplementary file 1 [file ijerph-17-05356-s001.pdf]

International Journal of
Environmental Research and Public Health

MDPI

Supplemental File S1: Articles excluded and reasons

No Randomized controlled trials:

1. Lee, K.B.; Kim, M.K.; Jeong, J.R.; Lee, W.H. Reliability of an Electronic Inspiratory Loading Device for Assessing Pulmonary Function in Post-Stroke Patients. *Med. Sci. Monit.* **2016**,*22,* 191–196. doi:10.12659/msm.895573.
2. Ramos, S.M.; Silva, D.M.; Buchaim, D.V.; Buchaim, R.L.; Audi, M. Evaluation of Respiratory Muscular Strength Compared to Predicted Values in Patients with Stroke. *Int. J. Environ. Res. Public Health* **2020**, *17*, 1091.
3. Yoo, Hyun-Joon MD.; Pyun, Sung-Bom MD, PhD Re: The Efficacy of Bedside Respiratory Muscle Training in Patients with Stroke. *Am. J. Phys. Med. Rehabil.* **2019**, *98*, 76–77. doi: 10.1097/PHM.0000000000001089.
4. Jo, M.R.; Kim, N.S. Combined respiratory muscle training facilitates expiratory muscle activity in stroke patients. *J. Phys. Ther. Sci.* **2017**, *29*, 1970.

No Results:

5. Jung, J.H.; Kim, N.S. The effect of progressive high-intensity inspiratory muscle training and fixed high-intensity inspiratory muscle training on the asymmetry of diaphragm thickness in stroke patients. *J. Phys. Ther. Sci.* **2015**, *27*, 3267–3269.

Intervention was not IMT/EMT:

6. Kim, C.B.; Shin, J.H.; & Choi, J.D. The effect of chest expansion resistance exercise in chronic stroke patients: A randomized controlled trial. *J. Phys. Ther. Sci.* **2015**, 27, 451–453.
7. Lee, M.H.; Jang, S.H. The effects of the neck stabilization exercise on the muscle activity of trunk respiratory muscles and maximum voluntary ventilation of chronic stroke patients. *J. Back Musculoskelet Rehabil.* **2019**, *32*, 863–868.
8. Slupska, L.; Halski, T.; Żytkiewicz, M.; Ptaszkowski, K.; Dymarek, R.; Taradaj, J.; & Paprocka-Borowicz, M. *Advances in Pulmonary Medicine: Research and Innovations*, Springer, Berlin, Germany, 2019, pp. 81–91.
9. Seo, K.C.; Lee, H.M.; & Kim, H.A. The Effects of Combination of Inspiratory Diaphragm Exercise and Exspiratory Pursed-lip Breathing Exercise on Pulmonary Functions of Stroke Patients. *J. Phys. Ther. Sci.* **2013**, *25*, 241–244. doi:10.1589/jpts.25.241.
10. Shen, S.C.; Nachalon, Y.; Randall, D.R.; Nativ-Zeltzer, N.; & Belafsky, P.C. (2019). High elevation training mask as a respiratory muscle strength training tool for dysphagia. *Acta. Otolaryngol* **2019**, *139*, 536–540.
11. Nam, C.W.; Park, Y.H.; Lee, J.H. Impact of cervical stabilization exercises and breathing retraining impact on the respiratory function of elderly stroke patients with hemiplegia: A randomized control trial. *J. Biosci. Bioeng.* **2015**, 7, 159–168.
12. Park, S.J.; Kim, S.H.; Min, K.O.The immediate effects of rib cage joint mobilization and chest wall stretch on muscle tone and stiffness of respiratory muscles and chest expansion ability in patients with chronic stroke. *J. Phys. Ther. Sci.* **2017**, *29*, 1960–1963.

Different language to English or Spanish:

13. YAMASHITA, K.; KIKUCHI, N.; & ITO, K. Effects of Expiratory Muscle Training on Respiratory Muscle Strength and Cough Intensity of Stroke Patients. *Rigakuryoho Kagaku* **2010**, *25*, 849–853. doi:10.1589/rika.25.849.
14. Back, W.C.; Kim, C.B. The Effects of Inspiratory Muscle Training with Chest Expansion on Pulmonary Function, Maximal Inspiratory Pressure, and Gait in Individuals with Stroke.Journal of Korea Proprioceptive Neuromuscular Facilitation Association. *Pnf Mov.* **2018**, *16*, 461–473.
15. Ma, D.; Wang, W. Clinical effects of respiratory training combined with conventional rehabilitation in hemiplegic patients. *Chin. J. Rehabil. Med.* **2016**, *31*, 1111–1116.

Reviews:

16. Veldema, J.; & Jansen, P. (2020). Ergometer training in stroke rehabilitation: Systematic review and meta-analysis. *Arch. Phys. Med. Rehabil.* **2020**, *101*, 674–689.
17. Liu J, Ren H, Yu Y, Chen Z, Xuu, K. Pulmonary Rehabilitation after Stroke. *Phys. Medizin. Rehabil. Kurortmed.* **2017**, *27*, 329–334.
18. Gomes-Neto, M.; Saquetto, M.B.; Silva, C.M.; Carvalho, V.O.; Ribeiro, N.; & Conceição, C.S. Effects of respiratory muscle training on respiratory function, respiratory muscle strength, and exercise tolerance in patients poststroke: A systematic review with meta-analysis. *Arch. Phys. Med. Rehabil.* **2016**, *97*, 1994–2001.

19. Martín-Valero, R.; Almeida, M.D.L.C.; Casuso-Holgado, M.J.; & Heredia-Madrazo, A. (2015). Systematic review of inspiratory muscle training after cerebrovascular accident. *Respir. Care* **2015**, *60*, 1652–1659.
20. Menezes KKP, Nascimento LR, Ada L, Polese JC, Avelino PR, Teixeira-Salmela LF. Respiratory muscle training increases respiratory muscle strength and reduces respiratory complications after stroke: A systematic review. *J. Physiother.* **2016**, *62*, 138–144.
21. Menezes, K.K.; Nascimento, L.R.; Avelino, P.R.; Alvarenga, M.T.M.; & Teixeira-Salmela, L.F. (2018). Efficacy of interventions to improve respiratory function after stroke. *Respir. Care* **2018**, *63*, 920–933.
22. Pollock, R.D.; Rafferty, G.F.; Moxham, J.; & Kalra, L. (2013). Respiratory muscle strength and training in stroke and neurology: A systematic review. *Int. J. Stroke* **2013**, *8*, 124–130.
23. Tong, Z.; Jun, Z. Guidelines for early rehabilitation of stroke in China. *Chin. J. Neurol.* **2017**, *50*, 405–412. DOI:10.3760/cma.j.issn.1006–7876.2017.06.002.

Protocols:

24. Kulnik, S.T.; Rafferty, G.F.; Birring, S.S.; Moxham, J.; Kalra, L. A pilot study of respiratory muscle training to improve cough effectiveness and reduce the incidence of pneumonia in acute stroke: study protocol for a randomized controlled trial. *Trials* **2014**, *15*, 1–10.
25. Wang, C.; Yu, L.; Yang, J.; Wang, R.W.; Zheng, Y.N.; Zhang, Y. Effectiveness of LiuZiJue Qigong versus traditional core stability training for poststroke patients complicated with abnormal trunk postural control: study protocol for a single-center randomized controlled trial. *Trials* **2020**, *21*, 1–11
26. Li, H.; Li, G.; Liu, G.; Zhang, Y. Liuzijue Qigong vs traditional breathing training for patients with post-stroke dysarthria complicated with abnormal respiratory control: study protocol of a single center randomized controlled trial. *Trials* **2018**, *19*, 335
27. Menezes, K.K.P.D.; Nascimento, L.R.; Polese, J.C.; Ada, L.; & Teixeira-Salmela, L.F. Effect of high-intensity home-based respiratory muscle training on strength of respiratory muscles following a stroke: A protocol for a randomized controlled trial. *Braz. J. Phys. Ther.* 2017, *21*, 372–377. doi:10.1016/j.bjpt.2017.06.017.

© 2020 by the authors. Licensee MDPI, Basel, Switzerland. This article is an open access article distributed under the terms and conditions of the Creative Commons Attribution (CC BY) license (http://creativecommons.org/licenses/by/4.0/).
